# Mesenchymal stem cell-derived exosomes improve motor function and attenuate neuropathology in a mouse model of Machado-Joseph disease

**DOI:** 10.1186/s13287-020-01727-2

**Published:** 2020-06-08

**Authors:** Hua-Jing You, Shu-Bin Fang, Teng-Teng Wu, Hongyu Zhang, Yu-Kun Feng, Xue-Jiao Li, Hui-Hua Yang, Ge Li, Xun-Hua Li, Chao Wu, Qing-Ling Fu, Zhong Pei

**Affiliations:** 1 Department of Neurology, The First Affiliated Hospital, Sun Yat-sen University; Guangdong Provincial Key Laboratory of Diagnosis and Treatment of Major Neurological Diseases, National Key Clinical Department and Key Discipline of Neurology, No. 58 Zhongshan Road 2, Guangzhou, 510080 Guangdong China; 2 Otorhinolaryngology Hospital, The First Affiliated Hospital, Sun Yat-sen University, No. 58 Zhongshan Road 2, Guangzhou, 510080 Guangdong China; 3 Department of Neurology, The Fifth Affiliated Hospital, Sun Yat-sen University, No. 52 Meihua Road East, Zhuhai, 519100 Guangdong China; 4 Guangdong Provincial Key Laboratory of Laboratory Animals, Guangdong Laboratory Animals Monitoring Institute, No. 11 Fengxin Road, Guangzhou, 510663 Guangdong China

**Keywords:** Machado-Joseph disease, Mesenchymal stem cell-derived exosomes, Motor function, Neuropathology

## Abstract

**Background:**

Machado-Joseph disease is the most common autosomal dominant hereditary ataxia worldwide without effective treatment. Mesenchymal stem cells (MSCs) could slow the disease progression, but side effects limited their clinical application. Besides, MSC-derived exosomes exerted similar efficacy and have many advantages over MSCs. The aim of this study was to examine the efficacy of MSC-derived exosomes in YACMJD84.2 mice.

**Methods:**

Rotarod performance was evaluated every 2 weeks after a presymptomatic administration of intravenous MSC-derived exosomes twice in YACMJD84.2 mice. Loss of Purkinje cells, relative expression level of Bcl-2/Bax, cerebellar myelin loss, and neuroinflammation were assessed 8 weeks following treatment.

**Results:**

MSC-derived exosomes were isolated and purified through anion exchange chromatography. Better coordination in rotarod performance was maintained for 6 weeks in YACMJD84.2 mice with exosomal treatment, compared with those without exosomal treatment. Neuropathological changes including loss of Purkinje cells, cerebellar myelin loss, and neuroinflammation were also attenuated 8 weeks after exosomal treatment. The higher relative ratio of Bcl-2/Bax was consistent with the attenuation of loss of Purkinje cells.

**Conclusions:**

MSC-derived exosomes could promote rotarod performance and attenuate neuropathology, including loss of Purkinje cells, cerebellar myelin loss, and neuroinflammation. Therefore, MSC-derived exosomes have a great potential in the treatment of Machado-Joseph disease.

## Background

Machado-Joseph disease (MJD), also known as spinocerebellar ataxia type 3 (SCA3), is the most common autosomal dominant hereditary ataxia worldwide, reaching 40–70% in Japan, China, and Portugal [1, 2]. Clinical symptoms include progressive cerebellar ataxia, ophthalmoplegia, dysarthria, spasticity, and peripheral neuropathy. It is caused by CAG repeat expansion in the *MJD1* gene, which encodes ATAXN3 protein. Mutant ATXN3 protein aggregates in neurons, forms nuclear inclusions, and disturbs the ubiquitin-proteasome pathway, leading to neurodegeneration, neuroinflammation, and brain atrophy especially in the cerebellar nuclei, brainstem, and basal ganglia [3, 4].

Given that there are currently no effective treatments for MJD, many attempts have been made to develop effective therapies to slow and stop this disease. Mesenchymal stem cells (MSCs) are multipotent stem cells that can differentiate into different cell types in the brain and release many potent factors. Since MSCs are easily obtained and expanded in vitro, MSC-based cell therapy has been extensively investigated in many neurological diseases, including MJD [5–9]. However, the clinical application of MSCs is hindered by side effects such as risks of oncogenicity and cellular embolism [10, 11]. Recently, increasing evidence has suggested that MSCs exert their therapeutic effects largely through paracrine secretion, such as exosomes. Exosomes are small vesicles of 30–100 nm in diameter that contain many cytokines and microRNAs [12]. MSC-derived exosomes have many advantages over MSCs, including higher efficiency of passing through the blood-brain barrier, longer half-life period, lower immunogenicity, higher stability, and easier storage and transportation conditions [13]. Their effects have been proven to be comparable with MSCs in different models of neurological diseases [14, 15].

In the present study, we aim to investigate whether MSC-derived exosomes can slow down the disease progression in a transgenic mouse model of MJD. We evaluated rotarod performance every 2 weeks and examined the loss of Purkinje cells, myelin loss, and neuroinflammation after exosomal treatment. We found that exosomes could improve rotarod performance, as well as attenuate neuropathology including loss of Purkinje cells, demyelination, and neuroinflammation. The present study suggests a promising potential of MSC-derived exosomes in the treatment of MJD.

## Methods

### Cell culture

Human urine cell-derived induced pluripotent stem cells (U-iPSCs) were donated by the Guangzhou Institute of Biomedicine and Health, Chinese Academy of Science, Guangzhou, China [16]. Human MSCs were generated from U-iPSCs according to the methods of our previous study and were passaged and cryopreserved at P10 at s density of 2 × 10^6^ per vial [16, 17]. The characteristics of iPSC-MSCs were the specific fibroblastic morphology; positive for CD105, CD73, CD146, CD144, and CD44; and negative for CD3, CD14, CD19, and CD45 (supplementary Fig. 1S). One vial of MSCs was thawed and cultured in two 150-cm^2^ cell culture plates and incubated with cell culture medium (CCM), as previously reported [16]. After 2–3 days, when the density of MSCs reached ~ 80%, the cells were further cultured in 25 150-cm^2^ cell culture plates and were incubated for 3–4 days. MSCs were then washed in phosphate-buffered saline (PBS) three times, and CCM was then replaced with chemically defined and protein-free (CDPF) medium as in our previous study [18], which contains CD-CHO medium (catalog number 10743-029, Gibco, USA), HT supplement (catalog umber 11067-030, Gibco, USA), l-glutamine (catalog number 25030-081, Gibco, USA), d-(+)-glucose (catalog number 50-99-7, Sigma, USA), non-essential amino acid (catalog number 11140-05, Gibco, USA), and Vitamin Solution (catalog number 11120-052, Gibco, USA). CDPF medium was replaced with fresh CDPF medium 6 h later, and MSCs were cultured for another 42 h. The supernatants from 6 to 48 h were collected and used for subsequent isolation of MSC-derived exosomes.

### Exosome isolation

**Fig. 1:**
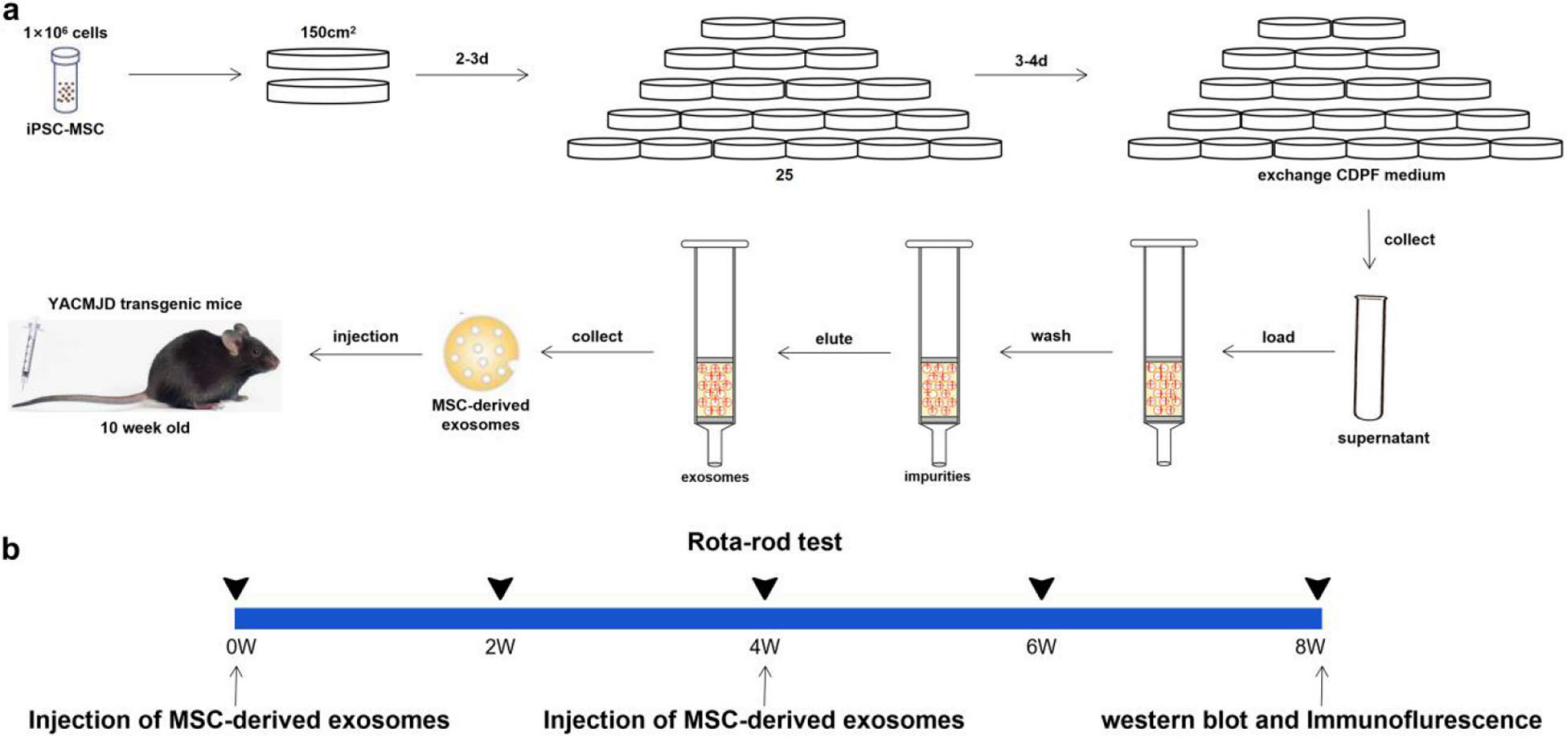
Longitudinal trial design to extract MSC-derived exosomes and assess their effects. **a** Schematic presentation including the proliferation of iPSC-MSCs, collection of supernatant, anion chromatography, and tail vein injection of MSC-derived exosomes. **b** Timeline to analyze motor function, western blot, and immunofluorescence staining after injection of MSC-derived exosomes twice in YACMJD84.2 mice

MSC-derived exosomes were isolated and purified through anion exchange chromatography as in our previous study [18]. Briefly, the chromatographic column was packed with 4 mL Q-sepharose and then washed with 12 mL equilibration buffer (50 mM phosphate buffer, 100 mM NaCl). Next, 150 mL supernatant containing MSC exosomes was loaded onto the column, which was washed with 40 mL wash buffer (50 mM phosphate buffer, 50 mM NaCl) to remove protein impurities. Bound material was then eluted with 1 mL elution buffer (50 mM phosphate buffer, 500 mM NaCl) for eight times. The concentrations of exosomes in these fractions were determined by Bradford Protein Assay, and the peak fractions were collected for dialysis. The final concentration of exosomes was determined by nanoparticle tracking analysis (Nanosight NS300; Malvern, UK). The MSC-derived exosomes were collected and stored at − 80 °C for further study (Fig. 1a).

### Identification of MSC exosomes

To positively identify exosomes, iPSC-MSC exosomes were denatured in SDS loading buffer for 5 min at 95 °C and then 10 μg of total protein was loaded and separated by SDS-PAGE on 10% gels. The proteins were transferred onto the PVDF membranes and stained with primary antibodies against CD63 (1:2000, catalog number Ab134045, Abcam, USA), Alix (1:5000, catalog number Ab186429, Abcam, USA), and TSG101 (1:1000, catalog number Ab125011, Abcam, USA) overnight at 4 °C after blocking with 4% skim milk. Then, the membranes were stained with goat anti-rabbit IgG HRP-conjugated antibody (1:2000, catalog number cw0103s, CWBIO) for 1 h and detected with Immobilon™ Western Chemiluminescent HRP substrate (Millipore, Billerica, MA, USA).

### Animals

All experimental procedures were in accordance with the guidelines of the Animal Research Committee of The First Affiliated Hospital, Sun Yat-sen University, Guangzhou, China, for the use and care of laboratory animals. YACMJD84.2 mice were generously provided by Professor Hong Jiang (Xiangya Hospital, Central South University, Changsha, China). The mice were generated in a C57BL/6 background strain and fed in a standard 12-h light/dark cycle with food and water ad libitum in the Laboratory Animals Monitoring Institute of Guangdong Province. Genotype identification was performed from DNA isolated from the tail tips using a Genomic DNA Mini Preparation Kit with Spin Column (Beyotime, China). Along with a pair of primers that amplified across CAG repeats in exon 10 of the human *MJD1* gene (MJD52 5′-CCAGTGACTACTTTGATTCG-3′/MJD25 5′-TGGCCTTTCACATGGATGTGAA-3′), Q84 hemizygosity was determined by quantitative real-time PCR using Premix Taq™ (Takara, Japan).

### Injection of MSC-derived exosomes

Tail vein injection was performed on 10-week-old YACMJDQ84.2 mice. MSC-derived exosomes (150 μL, approximately 1.5 × 10^10^ per dose) were transplanted at 0 and 4 weeks. Motor function and neuropathology were compared among YACMJD84.2 mice injected with MSC-derived exosomes (exosome-Tg, *n* = 8), YACMJD84.2 mice without injection (Control-Tg, *n* = 8), and wild-type mice without injection (Control-Wt, *n* = 8) (Fig. 1b).

### Behavior test: rotarod test

Passive movement and motor coordination were evaluated with a rotarod apparatus (Leica). Three groups of mice were placed on the rotarod at an accelerated speed (4–40 rpm, 5 min), and the latency to fall was recorded. The mean latency to fall over three trials was calculated. The rotarod test was performed every 2 weeks at a similar time point.

### Tissue preparation

After intraperitoneal injection of chloral hydrate, all mice were intracardiacally perfused with 50 mL of PBS (pH 7.4) at 18 weeks. One hemisphere was removed sagittally and fixed with cold 4% paraformaldehyde/PBS overnight at 4 °C. The hemispheres were then dehydrated with 20% and 30% sucrose/PBS successively at 4 °C and embedded in the Tissue-Tek OCT compound (Sakura Finetek Japan, Tokyo, Japan). Finally, each cerebellum was sagittally or coronally sectioned with a frozen microtome (Leica) and stored at − 80 °C until further use.

### Immunohistochemistry

After rewarming, the sections were incubated in 0.3% Triton™ X-100 and Immunol Staining Blocking buffer (Beyotime, China) for 1 h at room temperature. The sections were then incubated overnight at 4 °C with the following primary antibodies: mouse anti-calbindin D28K (1:500, catalog number C9848, Sigma-Aldrich, USA), mouse anti-ATXN3 (1H9) (1:200, catalog number MAB5360, Millipore, USA), rabbit anti-glial fibrillary acidic protein (GFAP) (1:200, catalog number ab48050, Abcam, USA), and rat anti-myelin basic protein (MBP) (1:200, catalog number ab7349, Abcam, USA), followed by incubation with the following secondary antibodies: goat-anti mouse IgG (H+L), F(ab’)2 fragment (Alexa Fluor® 488 conjugated) antibody (1:300, catalog number 4408S, Cell Signaling, USA), goat-anti mouse IgG (H+L), F(ab’)2 fragment (Alexa Fluor® 555 conjugated) antibody (1:300, catalog number 4409S, Cell Signaling), goat-anti rabbit IgG (H+L), F(ab’)2 fragment (Alexa Fluor® 488 conjugated) antibody (1:300, catalog number 4412S, Cell Signaling), and goat-anti rat IgG (H+L), F(ab’)2 fragment (Alexa Fluor® 488 conjugated) antibody (1:300, catalog number 4416S, Cell Signaling) for 1 h at room temperature. Finally, DAPI was used to identify the nuclei. Immunofluorescence images were obtained with a confocal laser scanning microscope (Leica) or a two-photon confocal laser scanning microscope (Leica).

### Quantification of Purkinje cells

Quantification of calbindin-positive Purkinje cells was performed by scanning three coronal sections in each group under the fluorescence microscope (*n* = 4). For each section, three fixed concave lobules were selected [19]. Linear density of calbindin-positive Purkinje cells along the cerebral cortex was determined as the number of Purkinje cells/mm [20]. The average cell numbers and the relative ratio of calbindin-positive Purkinje cells compared with YACMJD84.2 mice without exosomal injection were calculated using ImageJ (National Institutes of Health, Bethesda, MD, USA).

### Quantification of fluorescence intensities

Fluorescence intensities of MBP were analyzed by scanning three fixed cerebellar lobules in three coronal sections of each group [21]. The signal intensity of MBP in granular layers and white matter/0.25mm^2^ was calculated using ImageJ. Signal intensity in wild-type mice was used as the reference to calculate the relative ratio in the other two groups. The number of nuclear inclusions (1H9) and GFAP-positive fibers of the surrounding astrocytes/0.25 mm^2^ in cerebellar nuclei was also evaluated similarly using ImageJ.

### Western blot

Protein lysates from the cerebellums were obtained using RIPA Lysis and Extraction Buffer (Thermo Scientific, Rockford, IL, USA) containing protease inhibitors (1:100, PMSF, Solarbio, China). After sonication and centrifugation at 12000*g* for 15 min at 4 °C, the supernatants were collected. The protein concentration was determined with the Pierce™ BCA Protein Assay Kit (Thermo Scientific) and adjusted to the same concentration. All samples were stored at − 80 °C. Fifty micrograms of total proteins per sample was separated by 12.5% SDS-PAGE gels (PAGE Gel Fast Preparation Kit, catalog number PG113, Epizyme, China) according to the manufacturer’s protocol. Then, the proteins were transferred onto the PVDF membranes (Merck Millipore, Carrigtwohill, Ireland), followed by blocking with 5% skim milk for 1 h at room temperature. Rat anti-MBP (1:2000, catalog number ab7349, Abcam, USA), rabbit Bcl-2 (1: 1000, catalog number 3498S, Cell Signaling, USA), rabbit Bax (1: 2000, catalog number 14796S, Cell Signaling, USA), and mouse anti-ATXN3 (1H9) (1:1000, catalog number MAB5360, Millipore, USA) were incubated overnight at 4 °C. The membranes were then incubated with goat anti-rat IgG (H+L) HRP-linked antibody (1:1000, catalog number S0009, Affinity Biosciences, USA) for 1 h at 37 °C and were detected by using Immobilon™ Western Chemiluminescent HRP substrate (Millipore, Billerica, MA, USA).

### Statistical analysis

All experimental data are presented as the mean ± standard deviation/standard error of the mean for at least three independent experiments. The results were compared using one-way ANOVA. A *p* ≤ 0.05 was considered significant statistically.

## Results

### Characterization of MSC-derived exosomes

**Fig. 2:**
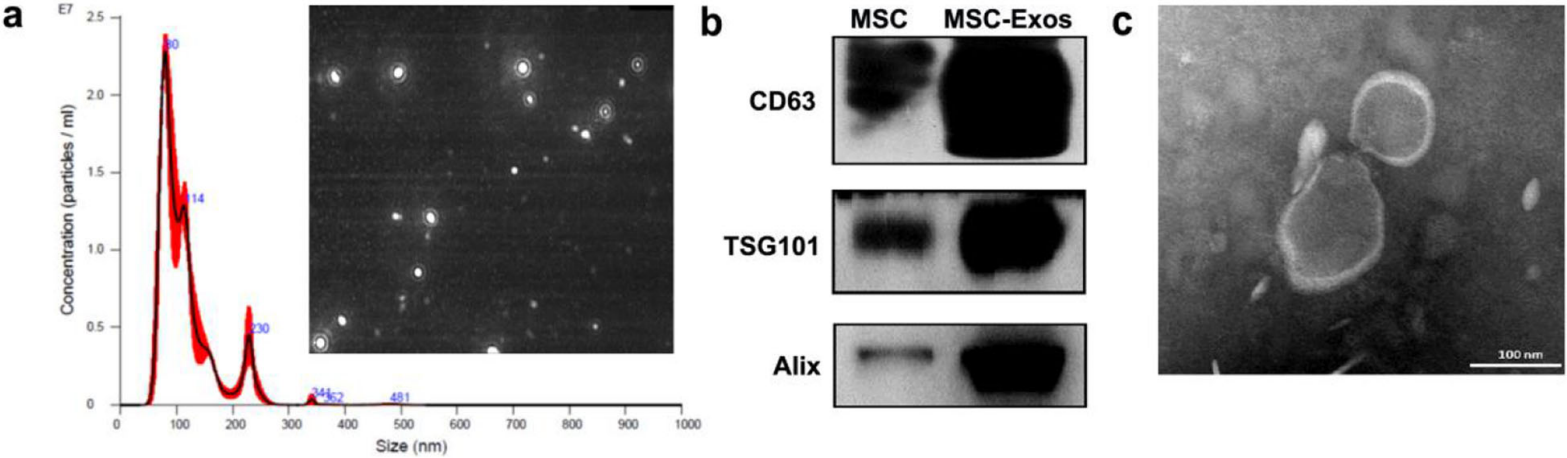
Characterization of exosomes derived from iPSC-MSCs. **a** The NTA of iPSC-MSC exosomes. **b** Compared with MSCs, the iPSC-MSC exosomes were strongly positive for CD63, TSG101, and Alix as determined by western blot. **c** The iPSC-MSC exosomes were bilipid-layered particles with a diameter of about 100 nm as shown by TEM

The total concentration and volumes of MSC-derived exosomes used in the current study were approximately 1.0 × 10^11^/mL and 200 μg/mL, as determined by nanoparticle tracking analysis and Bradford protein assay, respectively. The diameter of MSC-derived exosomes was approximately 100 nm (Fig. 2a). Compared with MSCs, western blot analysis revealed that MSC-derived exosomes were positive for specific exosomal markers, including CD63, TSG101, and Alix (Fig. 2b). Additionally, transmission electron microscopy showed that they were bilipid-layered particles (Fig. 2c). Taken together, these results demonstrated that high-quality MSC-derived exosomes could be isolated using anion exchange chromatography.

### MSC-derived exosomes improved motor function

To assess the effects of MSC-derived exosomes on motor function, exosomes were intravenously administrated into the tail vein of YACMJD84.2 mice. The rotarod test was used to evaluate motor function every 2 weeks until 8 weeks after treatment.

In the first 2 weeks, there was no significant difference in motor function among the normal controls (Control-Wt), YACMJD84.2 mice with exosomal treatment (Exosome-Tg), or those without exosomal treatment (Control-Tg). Four weeks after treatment, rotarod performance deteriorated in YACMJD84.2 mice without exosomal treatment (Control-Tg 261.837 ± 9.418 s). In contrast, the rotarod performance of YACMJD84.2 mice with exosomal treatment remained similar to normal control (Exosome-Tg 274.038 ± 5.608 s versus Control-Wt 279.050 ± 7.990 s). Six weeks after treatment, the deterioration of rotarod performance was more severe in YACMJD84.2 mice without exosomal treatment, compared with YACMJD84.2 mice with exosomal treatment and also with normal controls (Control-Wt 282.962 ± 7.656 s versus Exosome-Tg 279.675 ± 6.169 s versus Control-Tg 262.262 ± 9.597 s, *p* = 0.024). Eight weeks after treatment, rotarod performance deteriorated in YACMJD84.2 mice with exosomal treatment compared with normal controls (Exosome-Tg 263.487 ± 11.363 s versus Control-Wt 283.875 ± 11.487 s, *p* = 0.014). However, the difference in rotarod performance still remained significant between YACMJD84.2 mice with and without exosomal treatment (Exosome-Tg 263.487 ± 11.363 s versus Control-Tg 242.212 ± 20.538 s).

### MSC-derived exosomes attenuated apoptosis of Purkinje cells

**Fig. 3:**
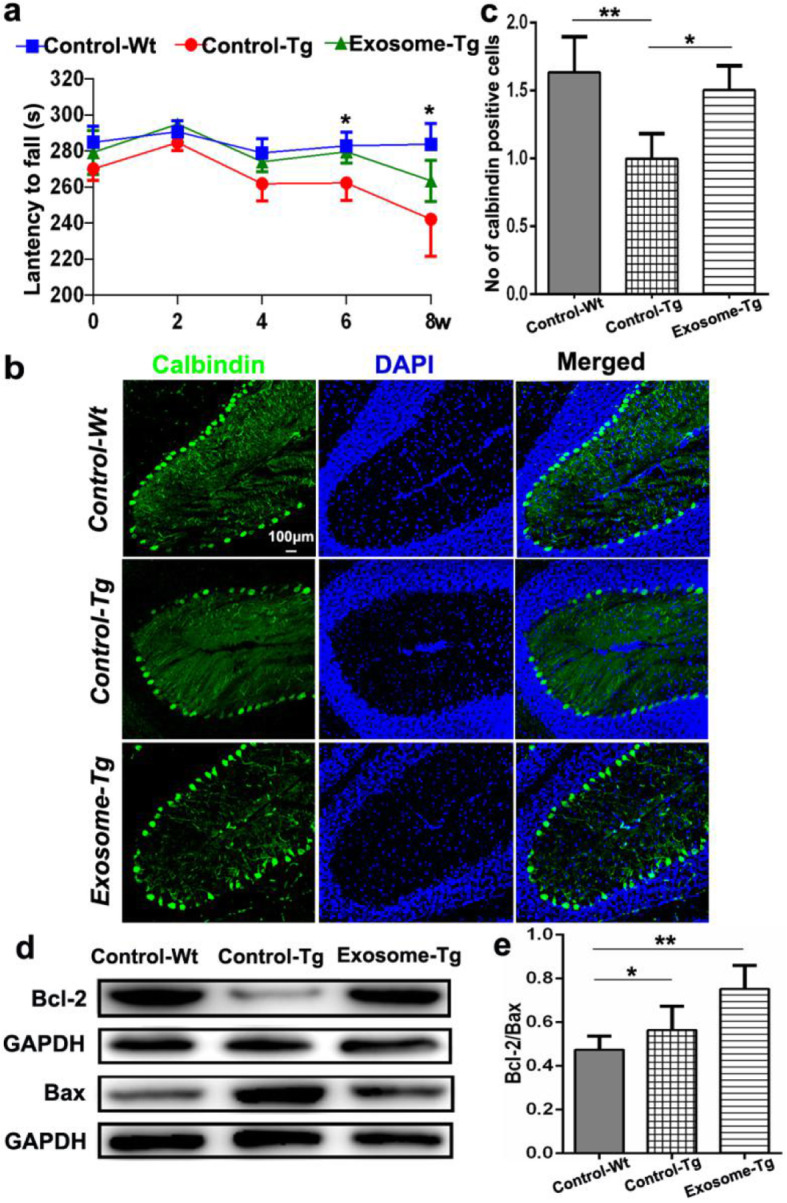
Injection of MSC-derived exosomes significantly improved motor function and rescued apoptosis of Purkinje cells. **a** Rotarod test was performed at an accelerated velocity in YACMJD84.2 mice with exosomal treatment (Exosome-Tg, *n* = 8), YACMJD84.2 (Control-Tg, *n* = 8), and wild-type littermates (Control-Wt, *n* = 8). Data was presented as mean ± SEM. **b**, **c** Quantitative and histological analysis of calbindin-positive cells in the sagittal cerebellar sections 8 weeks after injection (*n* = 4). The pictures were captured with a confocal laser scanning microscope at × 20 magnification. The scale bar was 100 μm. **d**, **e** Quantitative and western blot analysis of relative expression of Bcl-2/Bax protein (*n* = 4). One-way ANOVA analysis; *p ≤ 0.05, **p ≤ 0.01

Apoptosis of Purkinje cells, which is an important type of cell degeneration in MJD, was evaluated 8 weeks after exosomal treatment. Purkinje cells are responsible for cerebellar function. Consistent with the observations in both MJD patients and animal models, YACMJD84.2 mice exhibited a remarkable loss of Purkinje cells [22]. MSC-derived exosomes significantly attenuated the loss of Purkinje cells. The number of calbindin-positive Purkinje cells was 1.52 ± 0.20-fold higher in YACMJD84.2 mice with exosomal treatment than in those without exosomal treatment (Exosome-Tg 15.20 ± 1.73 versus Control-Tg 10.00 ± 1.89, *p* = 0.022, Fig. 3b, c). Although the number of Purkinje cells in YACMJD84.2 mice with exosomal treatment was slightly lower relative to normal controls, the difference was not significant (Control-Wt 16.20 ± 2.61 versus Control-Tg 15.20 ± 1.73, *p* > 0.05).

To explore the possible mechanism of the effect on Purkinje cells, the relative expression level of Bcl-2 and Bax was analyzed. The expression level of anti-apoptotic gene Bcl-2 in mice with exosomal treatment was higher than those without treatment, while the expression of pro-apoptotic gene Bax was just the opposite (Fig. 3d). Therefore, the relative ratio of Bcl-2/Bax in mice with exosomal treatment was significantly higher (Exosome-Tg 0.75 ± 0.054 versus Control-Tg 0.56 ± 0.054 versus Control-Wt 0.47 ± 0.031 s, *p* = 0.0073, Fig. 3e).

### MSC-derived exosomes attenuated myelin loss

**Fig. 4:**
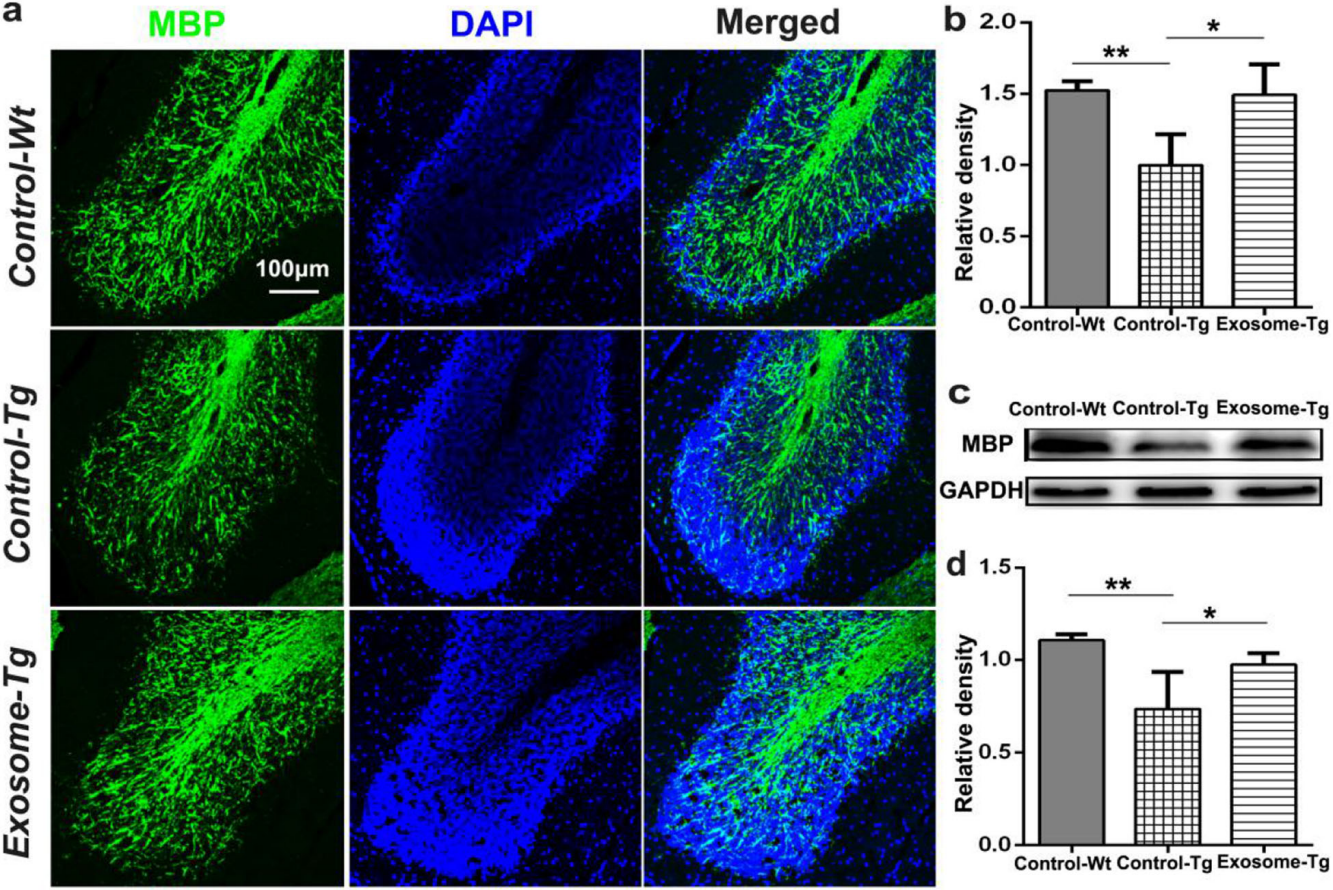
Injection of MSC-derived exosomes significantly attenuated hypomyelination in YACMJD84.2 mice. **a** Immunofluorescence images of MBP protein in the cerebellar lobules (*n* = 4). The pictures were captured with a two-photon confocal laser scanning microscope at × 25 magnification. The scale bar was 100 μm. **b** Quantitative analysis of MBP signal intensity (*n* = 4). One-way ANOVA analysis; *p ≤ 0.05, **p ≤ 0.01. **c** Western blot analysis of MBP expression among YACMJD84.2 mice with exosomal treatment (Exosome-Tg, *n* = 4), YACMJD84.2 mice without exosomal treatment (Control-Tg, *n* = 4), and wild-type littermates (Control-Wt, *n* = 4). **d** Quantitative analysis of relative MBP expression. One-way ANOVA analysis; *p ≤ 0.05, **p ≤ 0.01

The effect of MSC-derived exosomes on myelin loss was also evaluated 8 weeks after treatment. Myelin loss due to axonal inclusions and impaired axonal transportation are evident in MJD patients and animals [23, 24]. Myelin basic protein (MBP) is the main component of the myelin sheath, which surrounds axons in a molecular layer within the cerebellar white matter. The morphological changes and expression of MBP were assessed by immunofluorescence and western blot, respectively. Quantitative analysis of fluorescence intensity was performed following a previous report [25]. The relative intensity of MBP signals was 1.494 ± 0.211 and 0.999 ± 0 .217 in YACMJD84.2 mice with and without exosomal treatment, respectively (Fig. 4a, b). The results were further confirmed by western blot. The expression of MBP was also significantly higher in YACMJD84.2 mice with exosomal treatment than in those without exosomal treatment (Fig. 4c; Exosome-Tg 0.976 ± 0.060 versus Control-Tg 0.737 ± 0.199, *p* = 0.0185). Besides, signal intensity and expression of MBP in YACMJD84.2 mice with exosomal treatment were both slightly lower than those in normal controls, although these levels were not significant.

### MSC-derived exosomes attenuated neuroinflammation without reducing nuclear inclusions

**Fig. 5:**
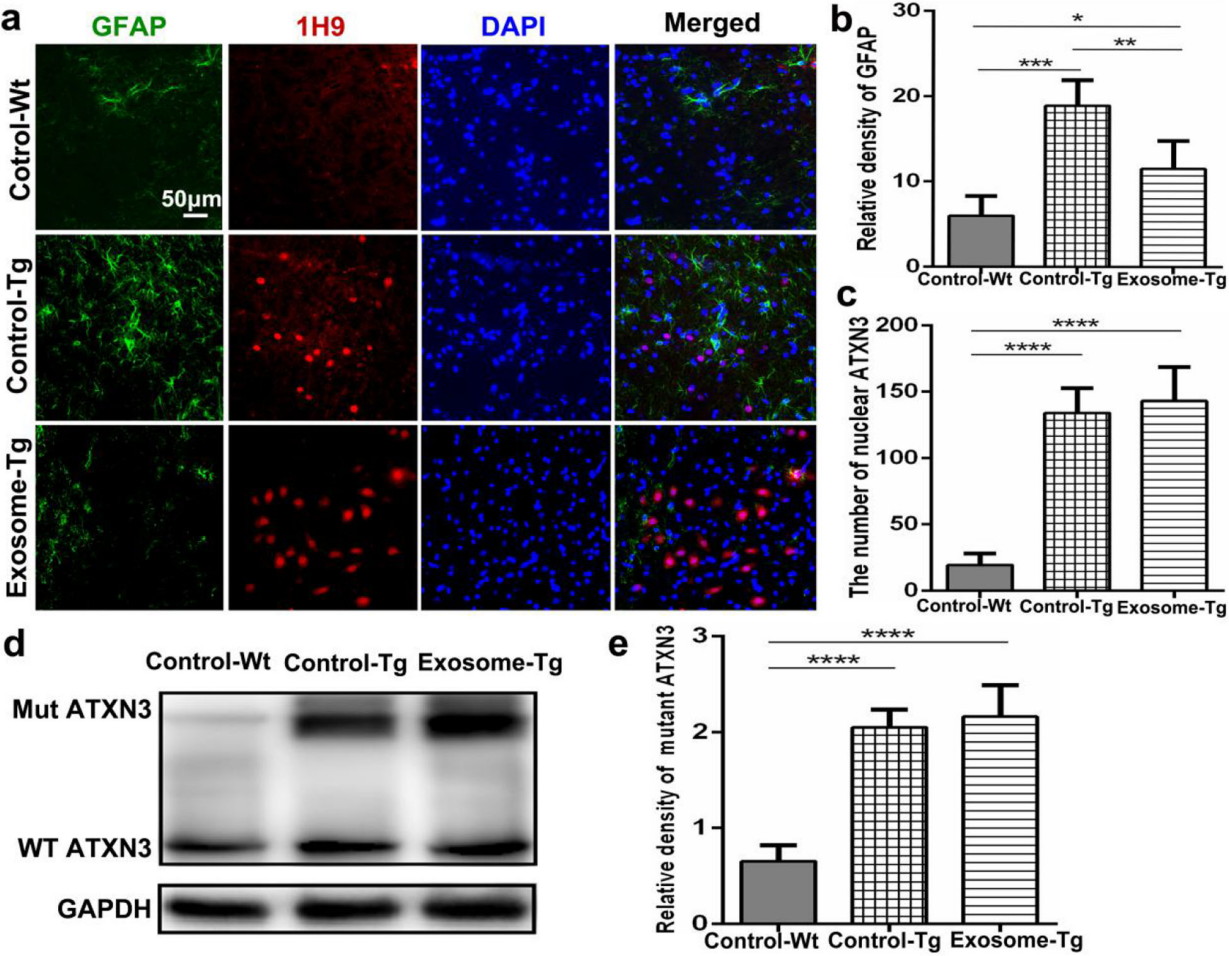
Injection of MSC-derived exosomes significantly reduced the activation of astrocytes. **a** Immunofluorescence images of astrocytes in pons among YACMJD84.2 mice with exosomal treatment (Exosome-Tg, *n* = 4), YACMJD84.2 mice without exosomal treatment (Control-Tg, *n* = 4), and wild-type littermates (Control-Wt, *n* = 4). The scale bar was 50 μm. **b** Quantitative analysis of GFAP signal intensity (*n* = 4). (**c**) Quantitative analysis of the number of nuclear inclusions stained by 1H9 (*n* = 4). **d** Western blot analysis of mutant (Mut) and wild-type (WT) ATXN3 expression among YACMJD84.2 mice with exosomal treatment (Exosome-Tg, *n* = 4), YACMJD84.2 mice without exosomal treatment (Control-Tg, *n* = 4), and wild-type littermates (Control-Wt, *n* = 4) in the cerebellum. **e** Quantitative analysis of mutant ATXN3 expression. One-way ANOVA analysis; *p ≤ 0.05, **p ≤ 0.01, ***p ≤ 0.001, ****p ≤ 0.0001

Inflammation and nuclear inclusions (NIs) associated with mutant ATXN3 in the brainstem are key components of MJD neuropathology [26, 27]. The effects of exosomal treatment on astrocytes and NIs were explored by immunofluorescence staining. The number of activated astrocytes was lower in YACMJD84.2 mice with exosomal treatment than in those without exosomal treatment, especially in the pons (Fig. 5a, b; Exosome-Tg 11.51 ± 3.281 versus Control-Tg 18.94 ± 2.983 versus Control-Wt 5.978 ± 2.320, *p* = 0.005). However, there was no significant difference in the number of NIs between these two groups (Fig. 5c). We further examined the expression of ATXN3 protein in the cerebellum by western blot. There was no difference in the expression levels of mutant ATXN3 between YACMJD84.2 mice with and without exosomal treatment (Fig. 5d, e).

## Discussion

MJD is a fatal polyglutamine neurodegenerative disease with no cure. In the present study, we evaluated the therapeutic potential of MSC-derived exosomes in a mouse model of MJD. Our result showed that MSC-derived exosomes can be isolated easily using anion exchange chromatography. In addition, MSC-derived exosomes can alleviate motor deficits and neuropathological changes in YACMJD84.2 mice. Thus, MSC-derived exosomes may have a great potential to be an effective therapy for MJD.

One of the more significant obstacles for exosome-based therapy is the difficulty with which to isolate sufficient numbers of exosomes. Ultracentrifugation is the most common method used to isolate exosomes; however, isolation of exosomes via ultracentrifugation is time-consuming [13]. Other disadvantages include possible sample contamination and damage to the architecture and components of exosomes. Compared with ultracentrifugation, the yield rate of exosomes isolated by anion exchange chromatography was nearly 2 times higher with only 20% of the time costing in our previous study [18]. Consistently, we achieved a high yield of good-quality exosomes from MSCs using anion exchange chromatography. The final concentration and volume, which were as high as 1.0 × 10^11^/mL and 200 μg/mL, respectively, may have a great potential to satisfy future requirements in clinical application.

The timing of intravenous administration is important and may influence the efficacy of exosomes. Similar to other polyglutamate diseases, the pathological alterations occur before disease onset in MJD patients and animal models [26, 28]. Therefore, pre-symptomatic treatment may be necessary. Indeed, MSC treatment failed to attenuate motor dysfunction and neuropathology when it was given after disease onset [29]. In the present study, a pre-treatment paradigm was adopted to test the possible efficacy of exosomes in YACMJD84.2 mice. YACMJD84.2 mice carry the yeast artificial chromosome (YAC) constructed with (CAG)_84_ and all necessary regulatory elements [30]. These mice are widely used in preclinical trials because they recapitulate typical motor phenotypes, as well as neuropathological features associated with MJD. In the present study, YACMJD84.2 mice developed motor deficits as evidenced by the decline in rotarod performance at the age of 14 weeks, which was consistent with a previous study [28]. Intravenous administration of exosomes significantly prolonged normal motor function for 4 weeks and attenuated motor deficits for an additional 2 weeks, when compared with YACMJD84.2 mice without exosomal treatment. Previously, mice receiving intravenous MSCs exhibited better motor function starting from 7 to 8 weeks after treatment. The beneficial effects of intravenous MSCs were also shown to last for 4–6 weeks [8, 31, 32]. By comparison, the protective effects of between exosomes and MSCs appear to be similar. Moreover, exosomes exerted effects starting from 4 weeks following treatment, which was 3 weeks faster than MSCs. These results suggest that exosomes may serve as a cell-free therapy to replace MSCs in the treatment of MJD. However, it should be noted that the protective effects decreased 8 weeks after treatment, although exosomes have a relatively long half-life, and suggests that a repetitive administration paradigm may be required to achieve a sustained alleviation of motor dysfunction [33].

Purkinje cells in the cerebellar cortex are key for motor coordination and send impulses that cooperate with deep cerebellar nuclei (DCN) and molecular effectors to regulate cerebellar neuronal circuits [34, 35]. Mutant ATXN3 expression induces degeneration of Purkinje cells, which in turn results in dyscoordination of MJD patients and animals [36]. Apoptosis is a major type of cell death in MJD. It is generally believed that the balance between pro-apoptotic Bax protein and anti-apoptotic Bcl-2 protein determine the fate of cells. Once activated, Bax oligomerizes at the mitochondrial outer membrane, induces permeability transition, and initiates apoptotic process. Positive immunoreactivity for Bax was found in the Purkinje cells of three MJD patients, indicating its possible pro-apoptotic mechanism in MJD [37]. In contrast, Bcl-2 blocks mitochondrial permeability transition and inhibits apoptotic process. In the present study, there was a noticeable loss of Purkinje cells and upregulated expression of Bax in the cerebellum of YACMJD84.2 mice. However, consistent with a previous study, exosomal treatment induced an increase in the relative expression of Bcl-2/Bax along with a decrease in cell loss, suggesting that exosome-induced high expression of Bcl-2/Bax may be responsible for the reduction of the Purkinje cell loss [38, 39].

Cerebellar afferent and efferent axons are responsible for the complicated connection between Purkinje cells, cerebellar nuclei, and the pons [40]. In the central nervous system (CNS), mutant ATAXN3 leads to hypomyelination, impairs axonal transportation, and induces death of interconnected neurons [22, 23, 30, 41]. MBP is the second most abundant protein and the only structural protein essential for myelination in the CNS [42]. MSC-derived exosomes have been shown to promote proliferation, differentiation, and migration of oligodendrocytes, which in turn rescued damaged axons and improved recovery of neurological function in different models of neurological diseases [43, 44]. Consistently, exosomal treatment significantly upregulated MBP signal intensity and expression in the cerebellum of YACMJD84.2 mice compared with those without treatment. Thus, exosomes may improve motor function, at least partially through rescuing myelin loss.

Gliosis is a prominent neuropathological feature in MJD brains [45]. Activated astrocytes, a key player of neuroinflammation, have been linked to neurodegeneration in MJD. Consistently, activated astrocytes were evident in YACMJD84.2 mice, while activation of astrocytes was significantly reduced in those with exosomal treatment, indicating the anti-inflammatory function of exosomes [46, 47]. Interestingly, mutant ATXN3 remained unchanged between mice with exosomes and those without exosomes. This observation is consistent with a previous report that MSC did not directly inhibit ATXN3 in a mouse model of MJD [9]. Thus, the protective mechanism by exosomes may not involve the inhibition of mutant ATXN3 protein. Future study is needed to reveal the anti-inflammatory mechanism of MSC-derived exosomes.

There are some limitations in our study. First of all, we did not examine the spinal cord of mice with MSC-derived exosomes. Given that the spinal cord can be affected in MJD, a future study on the efficacy of MSC-derived exosomes in the spinal cord will provide significant insights into the functions of exosomes in the treatment of MJD. In addition, other markers of Purkinje cells such as Zebrin II and cerebellin can be added in the future study to provide a better assessment of Purkinje cells. Moreover, therapeutic mechanisms are less explored. Thoughtful comprehensive mechanistic studies will be required to understand the therapeutic mechanisms of MSC-derived exosomes in MJD.

## Conclusion

We demonstrate that MSC-derived exosomes can improve motor function, reduce the loss of Purkinje cells, rescue the loss of myelin, and attenuate neuroinflammation. Collectively, MSC-derived exosomes are a promising therapeutic approach for MJD patients.

## Supplementary information

**Additional file 1: Figure S1.** Characterization of human induced pluripotent stem cell-derived mesenchymal stem cells. (a) Morphology of iPSC-MSCs (original magnification, 100×). (b) The MSC surface markers in iPSC-MSCs by flow cytometry. Cells were harvested at passage 9.

## Data Availability

The datasets generated and/or analyzed during the study are available from the corresponding authors on reasonable request.

## Abbreviations

MJD: Machado-Joseph disease
SCA3: Spinocerebellar ataxia type 3
iPSCs: Induced pluripotent stem cells
CCM: Cell culture medium
PBS: Phosphate-buffered saline
CDPF: Chemically defined and protein-free
MBP: Myelin basic protein
NIs: Nuclear inclusions
DCN: Deep cerebellar nucleus
YAC: Yeast artificial chromosome
CNS: Central nervous system
