# Supplementary material for: Mesenchymal stem cell-derived exosomes improve motor function and attenuate neuropathology in a mouse model of Machado-Joseph disease
Source: Stem Cell Res Ther. 2020 Jun 8;11:222. doi: 10.1186/s13287-020-01727-2 (PMC7278177; doi:10.1186/s13287-020-01727-2)
Supplement: Supplementary file 1 — Additional file 1: Figure S1. Characterization of human induced pluripotent stem cell-derived mesenchymal stem cells. (a) Morphology of iPSC-MSCs (original magnification, 100×). (b) The MSC surface markers in iPSC-MSCs by flow cytometry. Cells were harvested at passage 9. [file 13287_2020_1727_MOESM1_ESM.pdf]

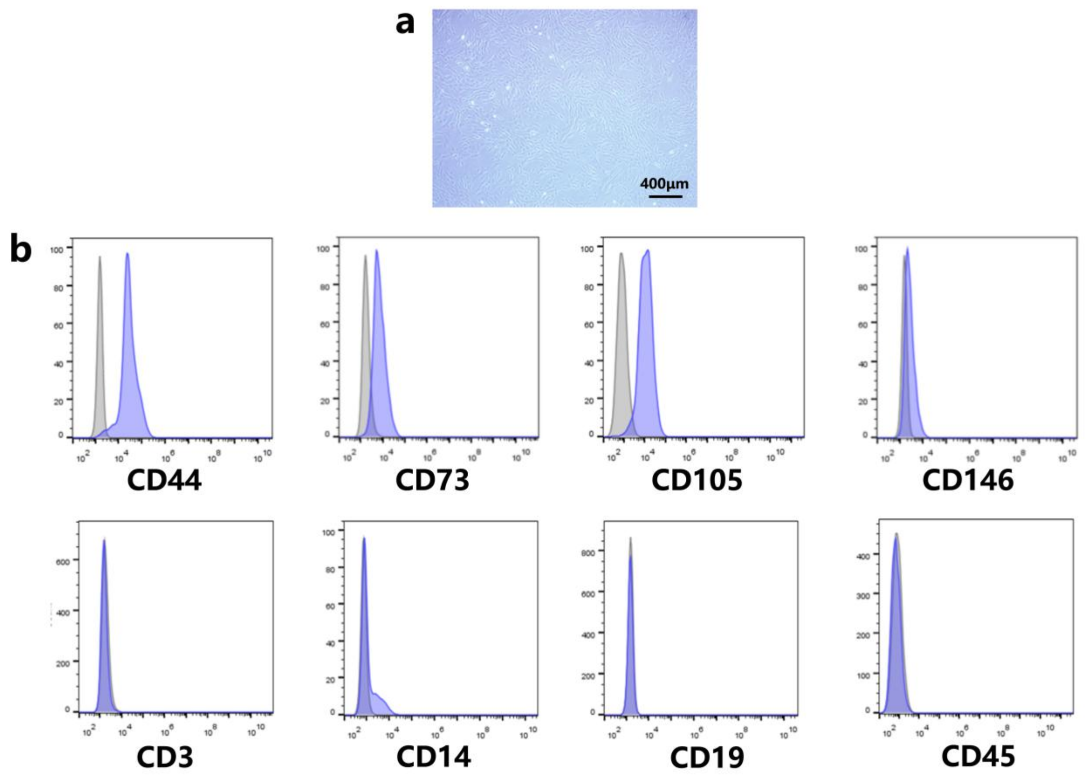

Fig. 1S Characterization of human induced pluripotent stem cell-derived mesenchymal stem cells. (a) Morphology of iPSC-MSCs (original magnification, 100 ×). (b) The MSC surface markers in iPSC-MSCs by flow cytometry. Cells were harvested at passage 9.
